# Strontium-baicalein coated β-tricalcium phosphate scaffold enhances diabetic bone regeneration via synergistic ROS scavenging and osteogenic activation

**DOI:** 10.1093/rb/rbag083

**Published:** 2026-04-26

**Authors:** Zhiqiang Liu, Yihao Wu, Chen Yang, Ruqi Wang, Linyi Hu, Sitong Hu, Xitao Wu, Jiang Chang, Jianfeng Ma, Jiandong Yuan

**Affiliations:** Department of Orthopaedics, The First Affiliated Hospital of Wenzhou Medical University, Wenzhou 325035, China; Institute of Stomatology, School and Hospital of Stomatology, Wenzhou Medical University, Wenzhou 325027, China; Zhejiang Engineering Research Center for Tissue Repair Materials, Wenzhou Institute, University of Chinese Academy of Sciences, Wenzhou 325000, China; Zhejiang Engineering Research Center for Tissue Repair Materials, Wenzhou Institute, University of Chinese Academy of Sciences, Wenzhou 325000, China; Orthopedic Institute, The First Affiliated Hospital, Suzhou Medical College, Soochow University, Suzhou 215000, China; Zhejiang Engineering Research Center for Tissue Repair Materials, Wenzhou Institute, University of Chinese Academy of Sciences, Wenzhou 325000, China; Department of Orthopaedics, The First Affiliated Hospital of Wenzhou Medical University, Wenzhou 325035, China; Zhejiang Engineering Research Center for Tissue Repair Materials, Wenzhou Institute, University of Chinese Academy of Sciences, Wenzhou 325000, China; Department of Orthopaedics, The First Affiliated Hospital of Wenzhou Medical University, Wenzhou 325035, China; Zhejiang Engineering Research Center for Tissue Repair Materials, Wenzhou Institute, University of Chinese Academy of Sciences, Wenzhou 325000, China; Institute of Stomatology, School and Hospital of Stomatology, Wenzhou Medical University, Wenzhou 325027, China; Department of Orthopaedics, The First Affiliated Hospital of Wenzhou Medical University, Wenzhou 325035, China

**Keywords:** diabetic bone, strontium-baicalein, coating, β-tricalcium phosphate

## Abstract

Conventional bone repair materials, such as β-tricalcium phosphate (β-TCP) scaffolds, are widely used in orthopedic applications due to their excellent biocompatibility and osteoconductivity. However, their regenerative efficacy is markedly compromised under diabetic microenvironments, as they lack the ability to counteract persistent oxidative stress and hyperglycemia-induced impairment of osteogenic activity. To address this limitation, we developed a strontium-baicalein (SrB) coated β-TCP scaffold (β-TCP@SrB) with integrated antioxidative and osteoinductive functions. In this system, baicalein provides robust cytoprotective and reactive oxygen species (ROS) scavenging effects under high-glucose (HG) conditions. Meanwhile, its polyphenolic structure enables strong interfacial adhesion to β-TCP and coordination-driven assembly with Sr^2+^ ions, which serve as potent osteogenic cues. *In vitro* studies demonstrated that β-TCP@SrB scaffolds effectively reduced excessive ROS accumulation and significantly enhanced osteogenic differentiation of HG-injured bone marrow mesenchymal stem cells, accompanied by activation of the Wnt signaling pathway. Furthermore, in a type 2 diabetic rat calvarial defect model, β-TCP@SrB scaffolds markedly promoted new bone formation compared with unmodified β-TCP scaffolds. Collectively, these results demonstrate a rational and clinically relevant surface-engineering strategy that integrates antioxidation and osteogenesis within a single bioactive interface, providing a promising approach for restoring bone regeneration in diabetes and potentially other metabolically compromised conditions.

## Graphical Abstract

**Graphical Abstract:**
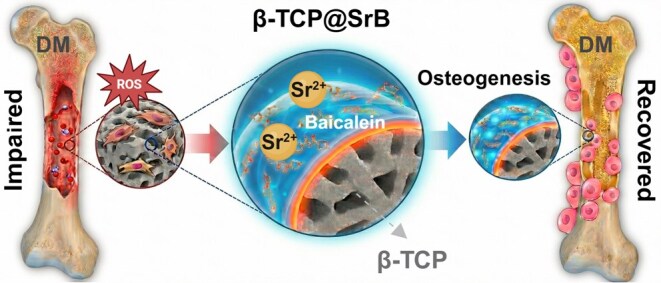

## Introduction 

The global burden of diabetes mellitus continues to rise, and this disease is accompanied by a markedly higher likelihood of fractures and bone defects in affected patients [1–4]. Although a variety of bone repair materials, characterized by excellent biocompatibility, osteoconductivity, and tunable manufacturing processes, have been developed, their efficacy remains highly dependent on the host’s intrinsic regenerative capacity [5–7]. β-tricalcium phosphate (β-TCP) scaffolds have been extensively studied as bone substitutes, largely because their bone mineral-like composition and favorable degradation behavior, which contribute to effective defect healing under healthy conditions [8, 9]. However, these advantages diminish markedly in the pathological milieu associated with diabetes [10]. Hyperglycemia-induced microenvironmental disturbances substantially interrupt normal tissue repair, leaving conventional scaffolds unable to provide adequate biological support [11, 12]. Compounding this challenge is the fact that currently available clinical bone graft substitutes are not engineered to adapt to the distinct biochemical and cellular perturbations associated with diabetes, resulting in frequent delays in bone healing and high rates of non-union [5]. Consequently, there is an urgent need to endow existing materials, such as β-TCP, with tailored functionalities that can actively counteract the aberrant diabetic microenvironment and restore their regenerative capabilities. Addressing this unmet need is essential for advancing therapeutic outcomes in diabetic patients suffering from bone defects.

Bone healing under diabetic conditions is disrupted by sustained hyperglycemia at multiple levels. Elevated glucose promotes nonenzymatic modification of tissue proteins and subsequent advanced glycation end products (AGEs) accumulation. Through interaction with RAGE, these glycation products trigger chronic inflammatory signaling, which in turn compromises osteoblast viability and osteogenic function [13]. Meanwhile, high glucose leads to overproduction of reactive oxygen species (ROS), through mitochondrial and enzyme-dependent pathways, thereby surpassing the buffering ability of the endogenous antioxidant system [14, 15]. Elevated ROS levels induce oxidative damage to bone marrow mesenchymal stem cells (BMSCs) and osteoblasts by impairing deoxyribonucleic acid (DNA) integrity, protein stability and membrane lipid integrity, ultimately diminishing their differentiation potential and capacity for matrix mineralization [16, 17]. Collectively, these inflammatory and oxidative insults disrupt the delicate balance of bone remodeling, enhancing osteoclastic activity while suppressing osteogenesis [14]. These combined disturbances help explain why bone healing is often compromised in diabetes, with delayed union and non-union occurring more frequently in affected patients [16, 18]. As such, functional strategies that can effectively mitigate oxidative stress, limit deleterious inflammation, and re-engage osteogenic differentiation are central to overcoming the biological barriers imposed by the diabetic microenvironment.

Natural polyphenolic compounds offer a promising avenue for addressing these pathological constraints as they possess substantial antioxidant and inflammation-modulating properties [19]. Baicalein, a representative flavonoid enriched with phenolic hydroxyl groups, exhibits potent radical-scavenging activity and has demonstrated the capacity to attenuate oxidative injury while promoting bone formation in various experimental models [20]. In addition to these pharmacological functions, polyphenols possess exceptional interfacial capabilities: their catechol and multi-hydroxyl structures enable a range of noncovalent interfacial forces such as hydrogen bonding and π–π interactions, making them well suited for surface functionalization. Notably, polyphenols also act as natural metal chelators. When co-present with metal ions, they undergo spontaneous coordination-driven assembly into metal-polyphenol networks (MPNs), in which metal ions serve as crosslinking nodes bridging multiple polyphenol ligands [9]. These MPNs can be deposited onto bone scaffold surfaces through mild, aqueous and highly controllable processes, enabling the creation of robust, uniform, and customizable coatings [21, 22].

Among commonly chelatable metal ions, magnesium (Mg^2+^), copper (Cu^2+^) and strontium (Sr^2+^) have been reported to play essential roles in bone regeneration [23]. Notably, Sr^2+^ ions have garnered particular attention due to their unique dual regulatory effects on bone metabolism [24–27]. On the one hand, Sr^2+^ activates calcium-sensing receptors expressed on osteoblasts and osteoblast-related cells, triggering downstream signaling pathways such as MAPK and Wnt, thereby promoting osteoblast proliferation, differentiation, and matrix synthesis. On the other hand, Sr^2+^ modulates bone resorption by inhibiting the formation and activity of osteoclasts, thus reducing overall bone resorptive activity. This dual mode of action, simultaneously enhancing bone formation while suppressing bone resorption, distinguishes Sr^2+^ from most other metal ions and underpins its clinical use in treating bone-loss conditions such as osteoporosis. Leveraging these advantages, incorporating Sr^2+^ into biomaterials or modifying material surfaces with Sr^2+^ has become an extensively applied strategy to enhance osteogenesis and support bone repair [28].

**Figure 1:**
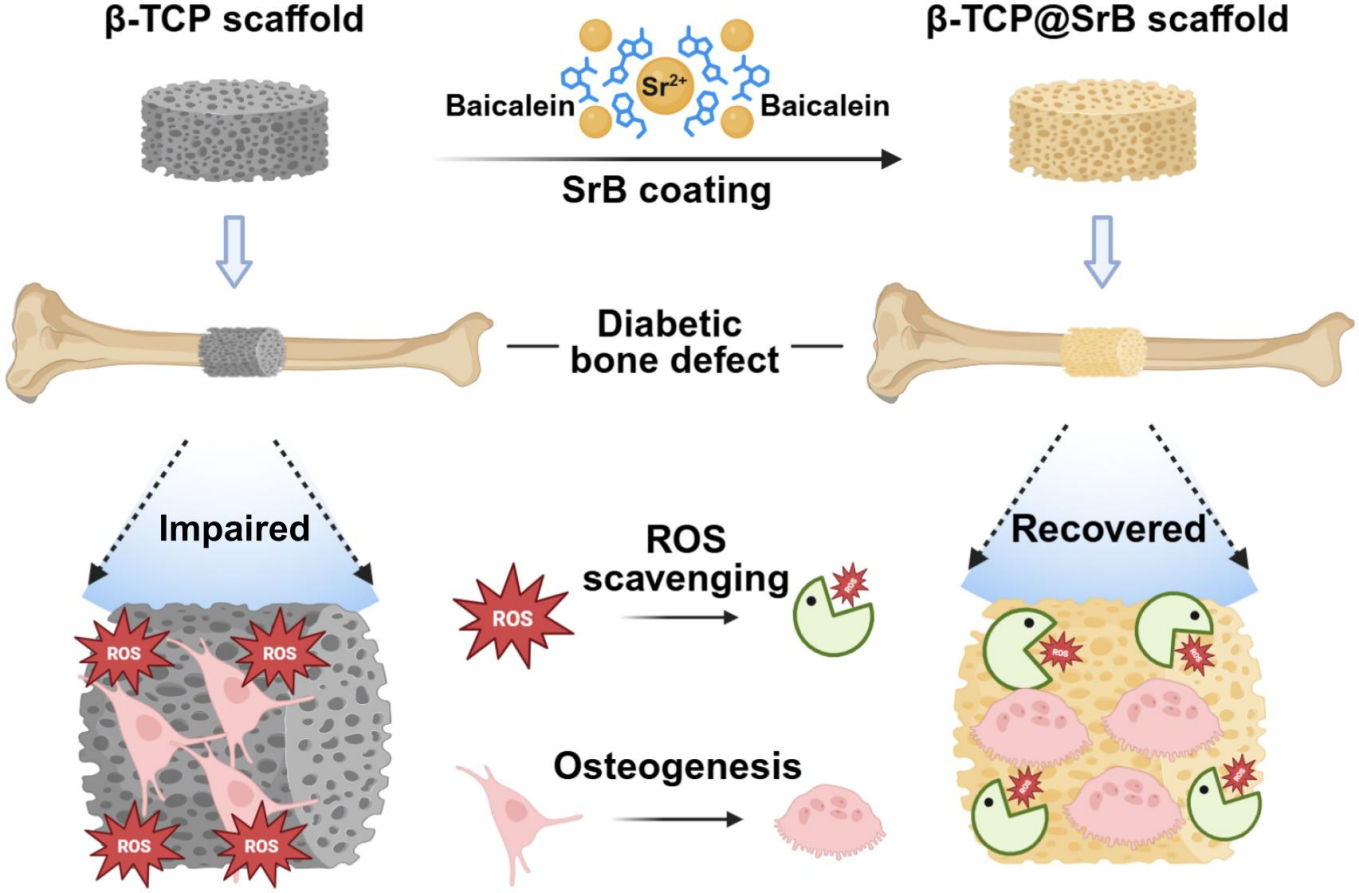
Schematic illustration of strontium-baicalein (SrB) coated β-tricalcium phosphate (β-TCP) scaffolds applied to diabetic bone repair. The SrB coating is constructed through coordination-driven integration of osteogenic Sr^2+^ ions with the ROS-scavenging flavonoid baicalein. This surface functionalization enables the scaffold to attenuate oxidative stress within the diabetic microenvironment while simultaneously enhancing osteogenic differentiation of bone marrow mesenchymal stem cells, thereby improving bone regeneration under hyperglycemic conditions. Figure was created using BioRender.com.

Building upon these considerations, we propose a targeted functionalization strategy for diabetic bone defect repair: the *in situ* construction of a strontium-baicalein (SrB) coating on β-TCP scaffolds (β-TCP@SrB) to enhance their regenerative performance under hyperglycemic conditions (Figure 1). This coating integrates the ROS-scavenging capacity of baicalein with the bone-modulating role of Sr^2+^, collectively forming a bioactive interface capable of counterbalancing the biochemical impediments of the diabetic microenvironment. To establish this platform, we first optimized the assembly conditions for the SrB coating and comprehensively characterized its morphology, chemical composition, coordination features and interfacial adhesion strength. We then assessed the antioxidative performance of β-TCP@SrB scaffolds and their ability to promote osteogenic differentiation under high-glucose (HG) conditions, alongside investigations into the underlying molecular mechanisms. Finally, the bone-healing performance of the modified scaffolds was subsequently evaluated *in vivo* in a type 2 diabetic (T2DM) rat calvarial defect model. Through this integrated experimental framework, the study aims to establish a rationally engineered coating strategy that converts conventional β-TCP into a functionally adaptive bone scaffold capable of addressing the complex challenges of diabetes-associated bone repair.

## Materials and methods

### Materials

All chemical reagents were purchased from Macklin (Shanghai, China), unless otherwise specified. β-tricalcium phosphate (β-TCP) powder was obtained from Kunshan Technology New Materials Co., Ltd. (Jiangsu, China).

### Fabrication of β-TCP scaffold

Porous scaffolds based on β-TCP were prepared through an SLA-assisted ceramic shaping process. Briefly, ceramic powder was dispersed in a light-curable resin matrix at a mass ratio of 3:2, yielding a printable precursor. The precursor was introduced into a stereolithography system (AUTOCERA-Ri, Beijing Ten Dimensions Technology, China), where the scaffold geometry was built by curing successive layers with ultraviolet (UV) radiation light at 12 mW cm^−2^ for 16 s per layer. After printing, the green scaffolds were fired at 1100°C to remove the organic component and convert them into porous ceramic β-TCP scaffolds.

### Preparation of β-TCP@SrB scaffold

β-TCP scaffolds were coated by exposing them to a mixed aqueous system containing baicalein and Sr^2+^, allowing surface-bound coordination to occur directly on the ceramic. For this process, 2 mL of baicalein solution at 0.06 mol L^−1^ was formulated with NaOH, whereas equal volume of SrCl_2_ solution (0.03 mol L^−1^) was directly formulated in deionized water. After equal-volume mixing of the two precursor solutions to form the coating medium, the scaffolds were introduced into the mixture and subjected to a 2 h reaction under 100 rpm agitation at 25°C. Following coating, the samples were washed thoroughly with deionized water to remove loosely bound components. The resulting specimens were denoted as β-TCP@SrB for one coating cycle. In optimization experiments, the alkalinity of the reaction system, the molar proportion of Sr^2+^ to baicalein, and the coating cycle number were examined. The condition adopted for the later experiments was identified according to CCK-8-based cytocompatibility results of neonatal rat femur-derived BMSCs.

### Characterization of β-TCP@SrB scaffold

Scaffold appearance was first inspected using an optical microscope (Olympus, Japan). Surface topography and microstructural details were further analyzed by scanning electron microscopy (SEM; JSM-7800F, JEOL, Japan), and elemental distribution was mapped with the attached energy-dispersive X-ray spectroscopy (EDS) detector. Surface chemical composition and bonding states were examined by X-ray photoelectron spectroscopy (XPS; PHI 5000 VersaProbe, ULVAC-PHI). X-ray diffraction (XRD) was used for phase identification (Bruker, Germany). UV-Vis absorption of baicalein and SrB was measured with a spectroscopic system (Agilent, USA). Interfacial attachment of the SrB layer on β-TCP was assessed by nano-scratch analysis (Wrexham, UK). The scaffolds were also subjected to mechanical testing with Instron system (USA).

The degradation behavior of the scaffolds *in vitro* was examined using a gravimetric method. Scaffolds were placed in neutral Tris-buffered saline and incubated at 37°C. At each scheduled time point (days 1, 3, 7 and 14), samples were retrieved, washed with deionized water, dried thoroughly and weighed. Degradation was expressed as the percentage decrease in mass compared with the initial weight.

### Radical-scavenging activity of β-TCP@SrB scaffold

#### 1,1-diphenyl-2-picryl-hydrazyl (DPPH) radical

For DPPH testing, a working solution was obtained by 50-fold dilution of a freshly prepared 1 mg mL^−1^ stock solution. Each scaffold specimen was immersed in 2 mL of this solution and incubated at 37°C for 30 min. Scavenging activity was estimated from the change in absorbance at 517 nm recorded on a UV–Vis spectrophotometer.

#### ABTS radical

For the ABTS decolorization assay, ABTS and potassium persulfate solutions (7.4 and 2.6 mmol L^−1^, respectively) were first combined at a 1:1 volume ratio and left in the dark at room temperature for 18 h to generate the radical species. Before use, the resulting solution was diluted 50 times. Scaffold samples were subsequently treated with 2 mL of this reagent at 37°C for 30 min, and radical removal was evaluated according to the absorbance decline at 734 nm.

#### Free ROS

The ROS-removing ability of the scaffolds toward superoxide anion (•O_2_^−^), hydrogen peroxide (H_2_O_2_), and hydroxyl radical (•OH) was determined separately using commercially available assay kits (Solarbio, Beijing, China). The assays were performed by following the protocols provided for each kit.

#### Intracellular ROS

To assess oxidative stress inside the cells, BMSCs were exposed to scaffold-conditioned indirect co-culture system in a high-glucose environment and then analyzed with 2′,7′-dichlorofluorescein diacetate (DCFH-DA). Briefly, cells were seeded into 24-well plates at a density of 1 × 10^4^ cells per well and maintained for 24 h, while the scaffolds were housed in Transwell chambers. After incubation, the cells were collected, replated and incubated with DCFH-DA following the supplier’s instructions. The fluorescence intensity of the probe oxidation product was used as an indicator of intracellular ROS accumulation.

### *In vitro* osteogenic differentiation ability of β-TCP@SrB scaffold

#### Alkaline phosphatase (ALP) staining

ALP histochemical staining was used as an indicator of early osteogenic differentiation. BMSCs were distributed into 12-well plates at 5 × 10^4^ cells per well, and scaffold-mediated stimulation was provided through a Transwell-based indirect culture setup. The cells were exposed to high-glucose osteogenic medium for 7 days. At the end of this period, fixation was carried out with 4% paraformaldehyde for 10 min, followed by staining with an ALP assay kit (Beyotime, Shanghai, China). Microscopic records of the staining results were obtained with an optical microscope (CKX53, Olympus, Japan).

#### Quantitative real-time polymerase chain reaction (RT-qPCR)

To analyze osteogenesis-related transcription, BMSCs were first plated in 12-well plates at 5 × 10^4^ cells per well and cultured for 14 days in a high-glucose osteogenic system with scaffold stimulation provided indirectly through Transwell inserts. After the induction period, total RNA was extracted from the cells and converted into complementary DNA (cDNA) by reverse transcription. The transcript levels of *Alpl*, osteocalcin (*Bglap*), and collagen type I alpha 1 chain (*Col1a1*) were then quantified by RT-qPCR using gene-specific primers. Relative expression values were determined by the comparative Ct approach, with glyceraldehyde-3-phosphate dehydrogenase (GAPDH) serving as the internal reference.

#### Alizarin Red S (ARS) staining

To assess late-stage osteogenic mineral deposition, BMSCs were maintained for 14 days in high-glucose osteogenic medium in a Transwell-based indirect co-culture system with scaffold samples. Cells were seeded in 12-well plates at 5 × 10^4^ cells per well before induction. At the end of the culture period, the samples were fixed in 4% paraformaldehyde, exposed to 2% (w/v) ARS solution under gentle agitation for 20 min, and then washed repeatedly until no excess dye remained. Mineralized nodules were subsequently identified by bright-field imaging.

#### Immunofluorescence (IF) staining

BMSCs were indirectly cultured with scaffolds in Transwell inserts under high-glucose osteogenic conditions. Cells were stained for Runt-related transcription factor 2 (RUNX2) after 7 days or for Osteocalcin (OCN) after 14 days. After fixation, permeabilization, blocking, and antibody incubation, nuclei were counterstained with 4',6-diamidino-2-phenylindole (DAPI). Fluorescence images were obtained by confocal microscopy (Nikon, Japan), and signal intensity was analyzed with ImageJ.

### RNA sequencing (RNA-seq) analysis

Transcriptomic changes were analyzed using cells cultured for 14 days in the presence or absence of β-TCP@SrB. Sample processing and sequencing were completed by Sangon Biotech Co., Ltd. (Shanghai, China). Briefly, messenger RNA (mRNA) containing poly(A) tails was separated from total RNA with oligo(dT)-linked magnetic beads, fragmented, and converted into complementary DNA (cDNA) by reverse transcription. Library construction then involved end polishing, 3′-adenylation, adaptor addition, fragment-size selection and PCR-based enrichment. After removal of adaptor contamination and poor-quality reads, the filtered sequences were mapped onto the reference genome. Subsequent analyses covered screening of differentially expressed genes (DEGs), Gene Ontology (GO) functional annotation, Kyoto Encyclopedia of Genes and Genomes (KEGG) pathway enrichment and Gene Set Enrichment Analysis (GSEA). Genes meeting the criteria of |log_2_(fold change)| > 1 and adjusted q < 0.05 in DESeq2 were considered differentially expressed. Volcano maps and clustered heatmaps were used for visualization.

### *In vivo* therapeutic effect of β-TCP@SrB scaffold

#### Type 2 diabetic rat calvarial defect model and scaffold implantation

The *in vivo* study employed a calvarial defect model in rats with type 2 diabetes. Compared with type 1 diabetes, this model more closely reproduces the metabolic imbalance and persistent inflammatory background that interfere with bone repair in diabetic conditions. All procedures received approval by the Ethics Committee of the Wenzhou Institute, Chinese Academy of Sciences (No. WIUCAS24101001). Six-week-old male Wistar rats were first maintained on a high-fat diet and then given intraperitoneal streptozotocin (STZ) to induce T2DM. Rats showing fasting blood glucose values of at least 16.7 mmol/L on two successive tests were classified as diabetic. Surgery was performed under isoflurane anesthesia with the animals lying supine. After a midline scalp incision, the periosteum was elevated to reveal the calvarium, and a circular defect of Ø5 mm was made with a surgical drill under continuous saline irrigation. The diabetic rats were then randomly assigned to blank, β-TCP and β-TCP@SrB groups.

#### Micro-computed tomography (micro-CT) analysis

For radiographic assessment, specimens from the calvarial defects were harvested at week 8 and preserved in 4% paraformaldehyde. Micro-CT scanning was then carried out on a SkyScan 1276 instrument (Bruker, Belgium). Three-dimensional reconstruction was completed with CTAn software, allowing the scaffold and newly formed bone to be displayed as distinct components. Morphometric analysis focused on the initial 5-mm defect region, from which bone mineral density (BMD), bone volume fraction (BV/TV), trabecular number (Tb.N) and trabecular separation (Tb.Sp) were calculated.

#### Hematoxylin and eosin (H&E) staining

For histological evaluation, specimens were first decalcified in EDTA and then processed routinely through ethanol dehydration, xylene clearing and paraffin embedding. Sections were cut to a thickness of 5 μm in either the sagittal or coronal orientation.

#### Masson’s trichrome staining

To further characterize repair-associated matrix formation, paraffin sections were processed with Masson’s trichrome staining, which was used to visualize collagen-rich extracellular matrix and tissue organization.

#### Immunohistochemical (IHC) staining

OCN and osterix (SP7) were detected on paraffin sections by immunohistochemistry. After citrate-based antigen retrieval, endogenous peroxidase was quenched and nonspecific binding was blocked with goat serum. The sections were then incubated with primary antibodies overnight at 4°C, followed by horseradish peroxidase-conjugated secondary antibodies for 1 h at 37°C. Positive signals were developed with 3,3′-diaminobenzidine and nuclei were counterstained with hematoxylin. The stained sections were dehydrated, mounted and observed by light microscopy.

### Statistical analysis

Results are shown as mean ± SEM. Group comparisons were performed in GraphPad Prism 10.0 using one-way ANOVA with Tukey’s test for multiple comparisons. A *P* value < 0.05 was considered significant. Significance levels are indicated as *P < 0.05, **P < 0.01 and ***P < 0.001.

## Results

### Optimization of SrB coating conditions

The strontium-baicalein (SrB) coating on β-TCP scaffolds was prepared using a one-pot assembly strategy. Key fabrication parameters, including solution pH, the molar ratio of Sr^2+^ ions to baicalein, reaction time, and precursor concentration, were systematically investigated. Baicalein solubility increased with increasing pH and reached complete dissolution at pH ≥ 9 (Supplementary Figure S1). Under this condition, immersion of β-TCP scaffolds in a mixed solution of baicalein and Sr^2+^ resulted in the formation of a visually apparent orange-brown coating on the scaffold surface. The extent of coating deposition was strongly dependent on the initial feeding ratio of Sr^2+^ to baicalein, with more pronounced and uniform coatings observed at molar ratios of 1:2 and 1:3 (Supplementary Figure S2). In addition, the coating thickness could be effectively regulated by adjusting the number of assembly cycles (Supplementary Figure S3). Within an appropriate thickness range, the SrB-coated scaffolds consistently exhibited favorable cytocompatibility (Supplementary Figure S4), confirming the feasibility of constructing bioactive SrB coatings on β-TCP scaffolds.

### Characterization of β-TCP@SrB scaffold

**Figure 2:**
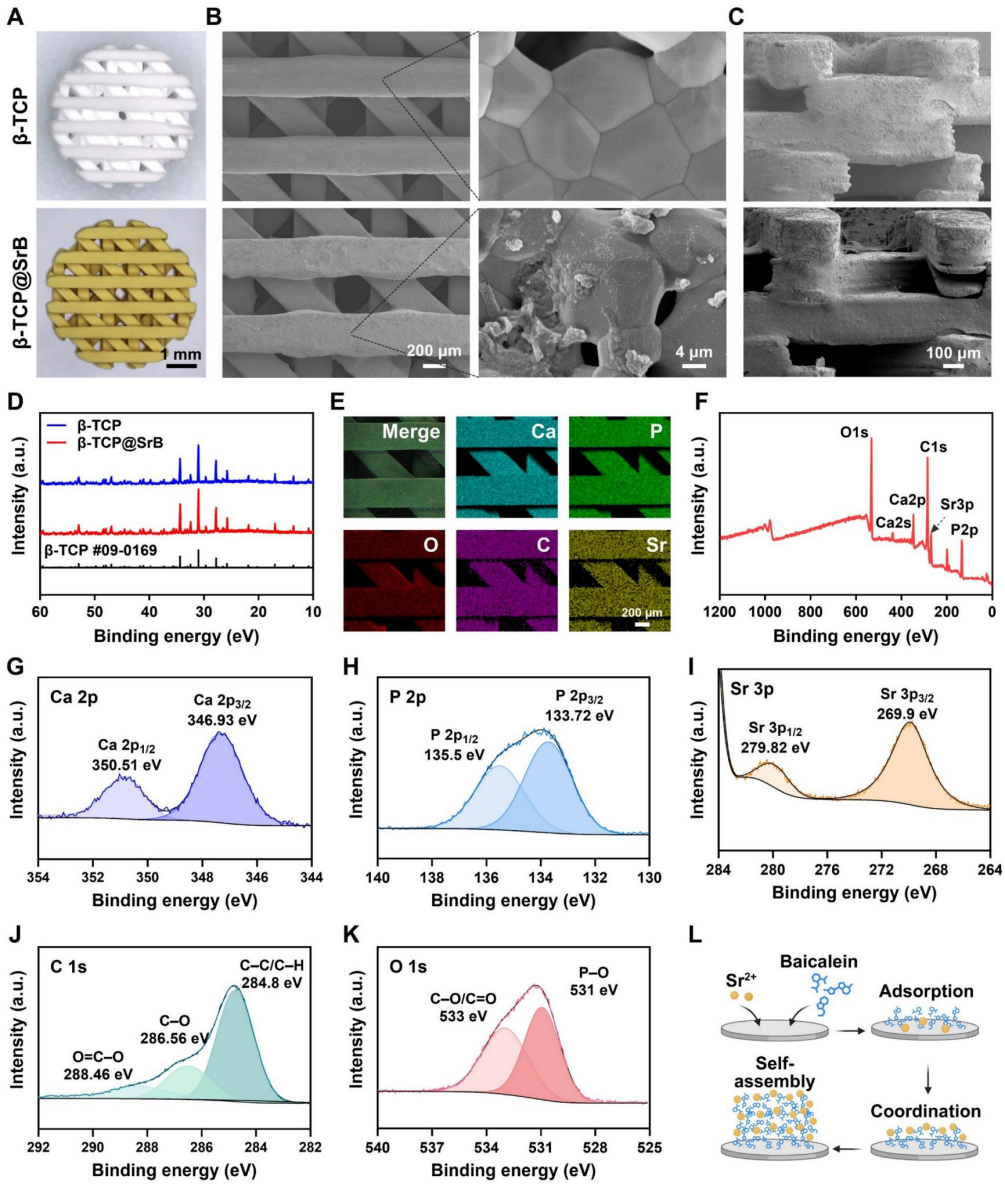
Structural and surface characterization of β-TCP@SrB scaffolds. (**A**) Representative photographs of unmodified β-TCP and β-TCP@SrB. (**B, C**) SEM characterization of scaffold morphology from the top view (B) and cross-sectional view (C). (**D**) XRD analysis of β-TCP and β-TCP@SrB. (**E**) EDS mapping of the coated scaffold. (**F**) Full-survey XPS spectrum of β-TCP@SrB. (**G–K**) High-resolution XPS spectra corresponding to Ca 2p, P 2p, Sr 3p, C 1s and O 1s, respectively. (**L**) Schematic representation of SrB coating formation on the β-TCP surface through coordination assembly. Panel L was generated using BioRender.com.

The physicochemical characteristics of the β-TCP@SrB scaffolds were subsequently analyzed in detail. As presented in Figure 2A, a brown SrB layer was distributed uniformly across the 3D-printed β-TCP scaffold surface. SEM observation further showed that the deposited layer was made up of irregular nanoscale particulates attached to β-TCP grains, resulting in a rougher surface than that of pristine β-TCP (Figure 2B). Importantly, the coating did not compromise the macroporous architecture or the overall 3D morphology of the scaffolds (Figure 2C). XRD analysis further demonstrated that SrB deposition did not alter the original crystalline phase of β-TCP, and no new diffraction peaks or phase transitions were observed after coating (Figure 2D).

EDS mapping showed that the coated scaffold surface contained calcium (Ca), phosphorus (P), oxygen (O), carbon (C) and strontium, consistent with successful deposition of the SrB layer (Figure 2E). The appearance of C and Sr signals, which were absent in pristine β-TCP, provided direct evidence for successful SrB deposition. Consistently, X-ray photoelectron spectroscopy (XPS) survey spectra detected the same elemental components (Figure 2F). High-resolution Ca 2p spectra exhibited characteristic peaks at 346.93 eV (Ca 2p_3/2_) and 350.51 eV (Ca 2p_1/2_), while P 2p spectra displayed doublets at 133.72 eV (P 2p_3/2_) and 135.5 eV (P 2p_1/2_), both originating from the β-TCP substrate (Figure 2G and H). In contrast, the Sr 3p spectrum showed distinct peaks at 279.82 eV (Sr 3p_1/2_) and 269.9 eV (Sr 3p_3/2_), indicating that Sr was present predominantly in the Sr^2+^ state and coordinated with oxygen-containing groups of baicalein (Figure 2I). The high-resolution C 1s spectrum exhibited three characteristic peaks at 284.8 eV (C–C/C–H), 286.56 eV (C–O) and 289.46 eV (O=C–O), corresponding to aromatic rings, hydroxyl groups, and carbonyl functionalities within the baicalein structure (Figure 2J). In the O 1s region, two major peaks were observed at 531 and 533 eV, corresponding respectively to P–O and Sr–O species, and to C=O and C–O bonds (Figure 2K), which further support the formation of coordination interactions between Sr^2+^ and baicalein at the β-TCP interface (Supplementary Figure S5).

The ultraviolet-visible (UV-Vis) spectrum of baicalein was characterized by two major absorption bands located at 248 and 273 nm. Upon coordination with Sr^2+^, these peaks exhibited a slight blue shift to 245 nm and 269 nm, respectively, accompanied by peak broadening and reduced intensity (Supplementary Figure S6). These spectral changes suggest altered π-electron delocalization in the conjugated system, which is consistent with Sr–O coordination involving the hydroxyl and carbonyl groups of baicalein. Collectively, the results support successful self-assembly of Sr^2+^-baicalein complexes on the β-TCP surface, forming a stable and uniform SrB coating.

### SrB coating preserved β-TCP scaffold integrity and enhanced its antioxidant activity

**Figure 3:**
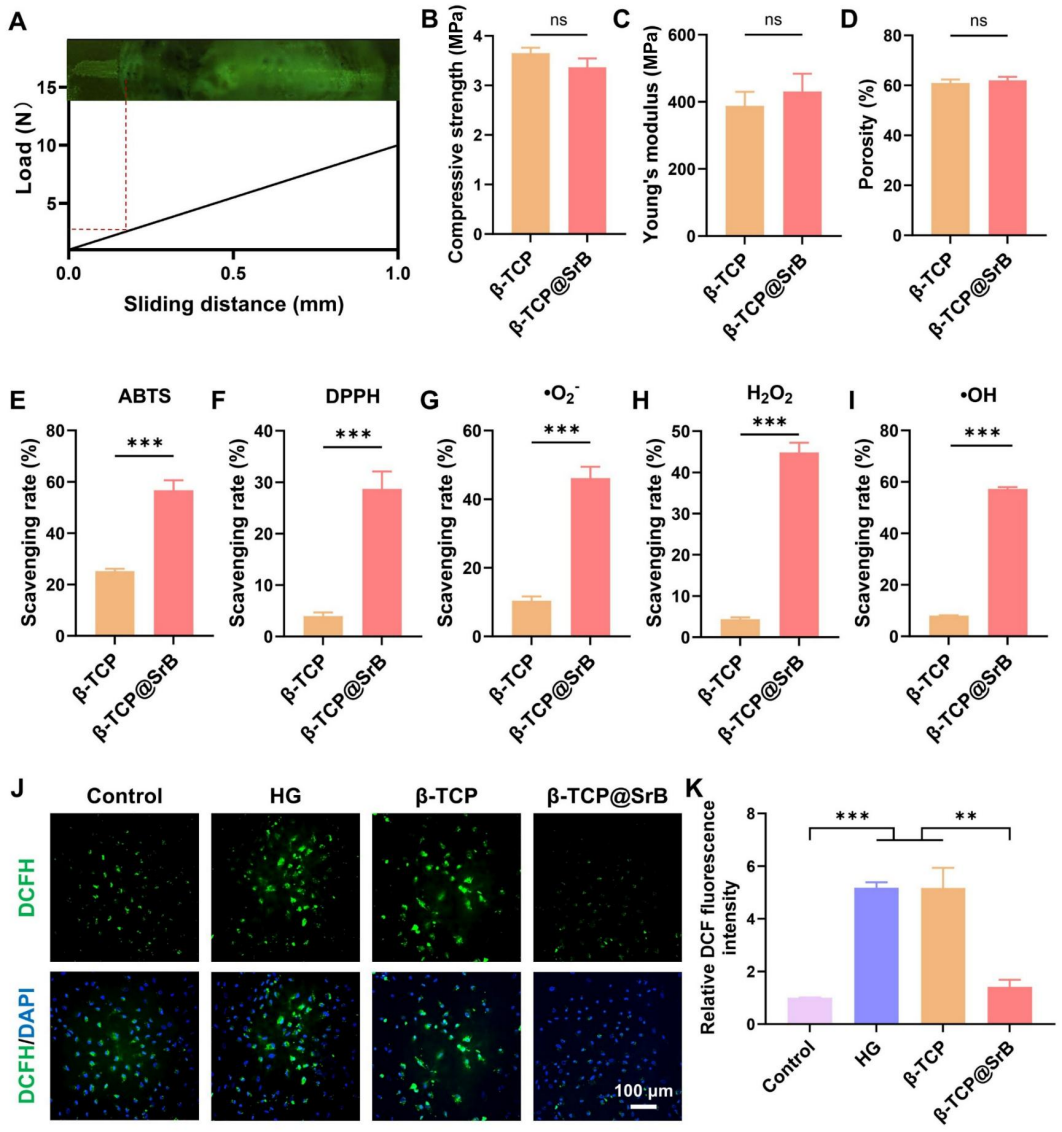
Evaluation of scaffold physicochemical characteristics and antioxidant activity *in vitro*. (**A**) Nano-scratch test showing the adhesion strength of the SrB interfacial layer. (**B–D**) Mechanical and structural parameters of β-TCP and β-TCP@SrB, including compressive strength, Young’s modulus and porosity. *n* = 4. (**E–I**) Scavenging capacities of the two scaffolds toward different radicals and ROS, including ABTS, DPPH, •O_2_^−^, H_2_O_2_, and •OH. *n* = 5. (**J, K**) Representative DCFH-DA fluorescence images of HG-stimulated BMSCs cultured with β-TCP or β-TCP@SrB and the corresponding quantification of intracellular ROS-associated fluorescence signals. *n* = 3.

Nano-scratch testing was initially performed to determine the adhesion strength of the SrB coating on β-TCP, since durable interfacial bonding is critical for the sustained function of surface-modified biomaterials. As shown in Figure 3A, the critical load for coating failure was around 2.5 N, indicating stable attachment of the SrB coating on the β-TCP surface. Subsequent compression testing, porosity measurements, and *in vitro* degradation assays revealed no significant differences in compressive strength, Young’s modulus, porosity, or degradation rate between β-TCP and β-TCP@SrB scaffolds (Figure 3B–D, Supplementary Figure S7). These results indicate that surface functionalization with SrB preserves the mechanical integrity and structural characteristics of the β-TCP scaffold.

By comparison, the SrB layer substantially improved the free-radical-scavenging performance of β-TCP scaffolds under *in vitro* conditions (Figure 3E–I). In detail, β-TCP@SrB scaffolds exhibited substantially higher scavenging efficiencies against both DPPH radicals (Supplementary Figure S8) and ABTS radicals (Supplementary Figure S9), achieving scavenging rates of 29% (Figure 3E) and 57% (Figure 3F), respectively. Both values clearly exceeded those measured for unmodified β-TCP scaffolds (3.98% for DPPH and 25.22% for ABTS). Moreover, β-TCP@SrB scaffolds demonstrated pronounced scavenging activity toward representative ROS, including •O_2_^−^, H_2_O_2_, and •OH. Compared with β-TCP scaffolds, the scavenging efficiencies of β-TCP@SrB were increased by approximately 4.44-fold, 10.24-fold and 7.06-fold for •O_2_^−^, H_2_O_2_ and •OH, respectively (Figure 3G–I).

Because hyperglycemic stress is typically associated with excessive intracellular ROS production, the ability of β-TCP@SrB to mitigate intracellular oxidative stress was further evaluated using DCFH-DA staining. As shown in Figure 3J, HG conditions induced a marked increase in intracellular ROS levels in BMSCs, which was not alleviated by pristine β-TCP. Under HG exposure, intracellular ROS remained high in BMSCs, whereas co-treatment with β-TCP@SrB greatly lowered this oxidative burden. As quantified in Figure 3K, the ROS signal in the β-TCP@SrB group was only 22.62% of that in the HG group, indicating effective suppression of intracellular oxidative stress.

Overall, the results suggest that the SrB layer remains structurally stable after surface modification and preserves the original architecture as well as the mechanical characteristics of the β-TCP scaffold, while endowing it with substantially enhanced antioxidant capacity and ROS-eliminating activity.

### SrB coating promoted the osteogenic performance of β-TCP in a HG microenvironment

**Figure 4:**
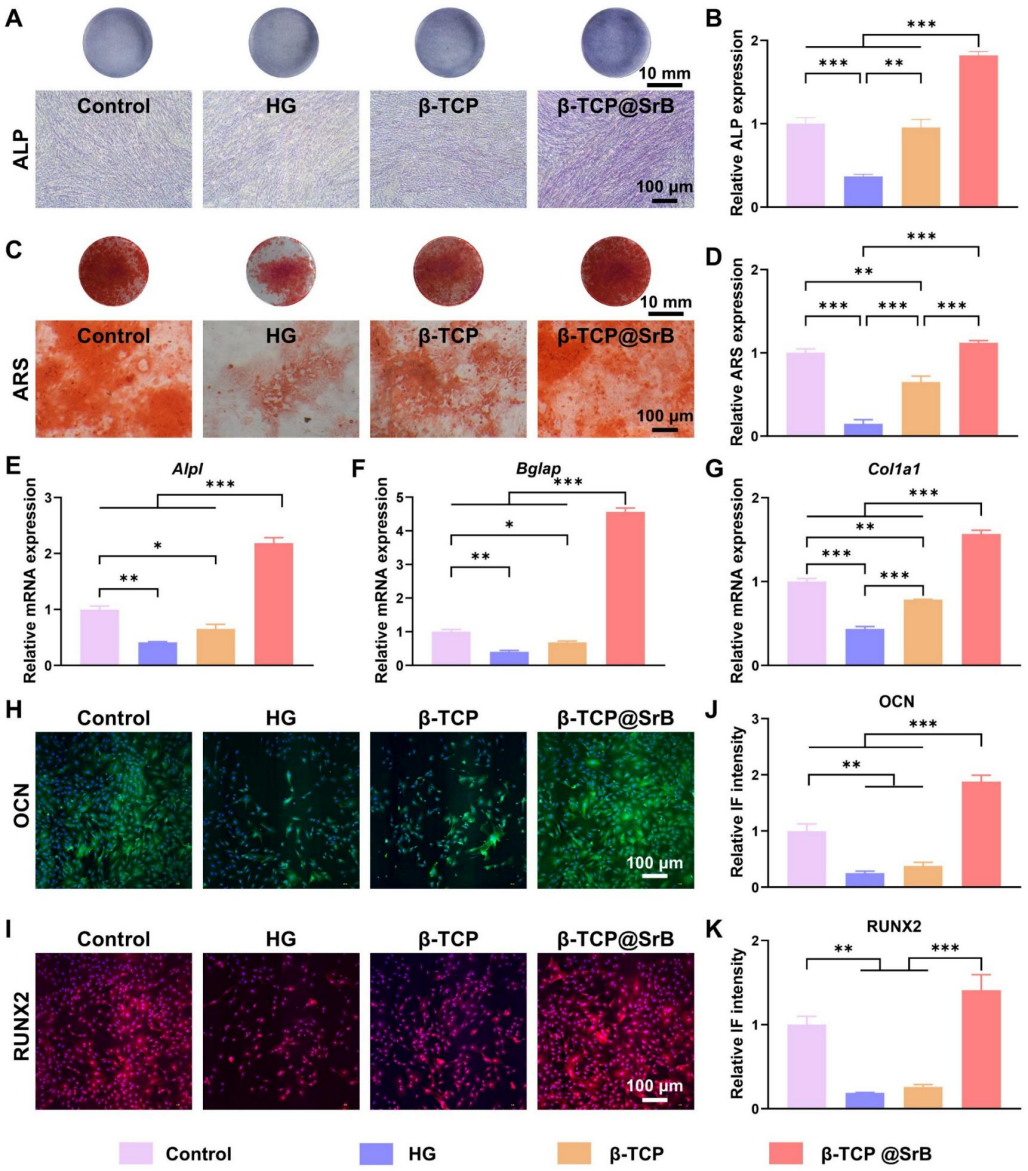
Osteogenic responses of BMSCs to β-TCP@SrB under HG culture. (**A, B**) Histochemical detection of ALP activity and its semiquantitative evaluation. *n* = 3. (**C, D**) Alizarin Red S staining for extracellular mineral formation, together with semiquantitative assessment. *n* = 3. (**E–G**) RT-qPCR analysis of osteogenesis-associated transcripts, namely *Alpl*, *Bglap*, and *Col1a1*. *n* = 3. (**H, J**) Fluorescent staining of OCN and measurement of signal intensity. *n* = 3. (**I, K**) Fluorescent staining of RUNX2 and measurement of signal intensity. *n* = 3.

After long-term HG stimulation, the bone-forming capacity of BMSCs is substantially weakened, and this dysfunction represents one of the central pathological barriers to bone repair in diabetes. To address this challenge, the osteogenic activation of HG-impaired BMSCs by β-TCP@SrB scaffolds was systematically evaluated. Alkaline phosphatase (ALP) staining revealed that HG stimulation significantly suppressed ALP expression in BMSCs, with the mean ALP level reduced to 36.93% of that observed in the Control group (Figure 4A and B). Treatment with pristine β-TCP substantially alleviated this suppression, whereas β-TCP@SrB treatment further enhanced ALP expression, increasing it to 181.96% relative to the Control group. Consistent trends were observed in Alizarin Red S (ARS) staining (Figure 4C), which reflects extracellular matrix mineralization. Among all groups, BMSCs cultured with β-TCP@SrB scaffolds exhibited the most pronounced calcium nodule formation, with relative mineralization levels reaching 1.12-fold and 7.58-fold of those in the Control and HG groups, respectively (Figure 4D).

Gene expression analysis by RT-qPCR further corroborated these findings. HG exposure significantly downregulated the expression of osteogenic marker genes, including *Alpl*, *Bglap* and *Col1a1* (Figure 4E–G). In contrast, β-TCP@SrB treatment effectively reversed this downregulation, resulting in 5.30-fold, 11.35-fold and 3.59-fold increases in *Alpl*, *Bglap* and *Col1a1* expression, respectively, compared with the HG group. Immunofluorescence (IF) staining of OCN and RUNX2 demonstrated similar patterns at the protein level (Figure 4H–K). HG conditions markedly reduced OCN (green fluorescence) and RUNX2 (red fluorescence) expression in BMSCs. Pristine β-TCP exhibited a modest restorative effect; however, this effect was not pronounced. In contrast, β-TCP@SrB treatment produced much stronger fluorescence signals than those seen in the Control group, suggesting effective recovery of osteogenic marker expression under HG conditions (Figure 4H and J). Fluorescence quantification showed that the β-TCP@SrB group exhibited OCN levels that were 1.88 times the Control level, 7.44-fold of the HG level and 4.93-fold of the β-TCP level (Figure 4J). RUNX2 followed the same trend, reaching 1.41-, 7.45- and 5.39-fold of the corresponding values in the Control, HG and β-TCP groups, respectively (Figure 4K).

Overall, the results suggest that β-TCP@SrB scaffolds can markedly rescue the osteogenic deficiency of BMSCs caused by HG exposure, and that the SrB coating plays a central role in re-establishing and promoting osteogenic function in a hyperglycemic environment.

### Transcriptomic analysis and mechanistic insights

**Figure 5:**
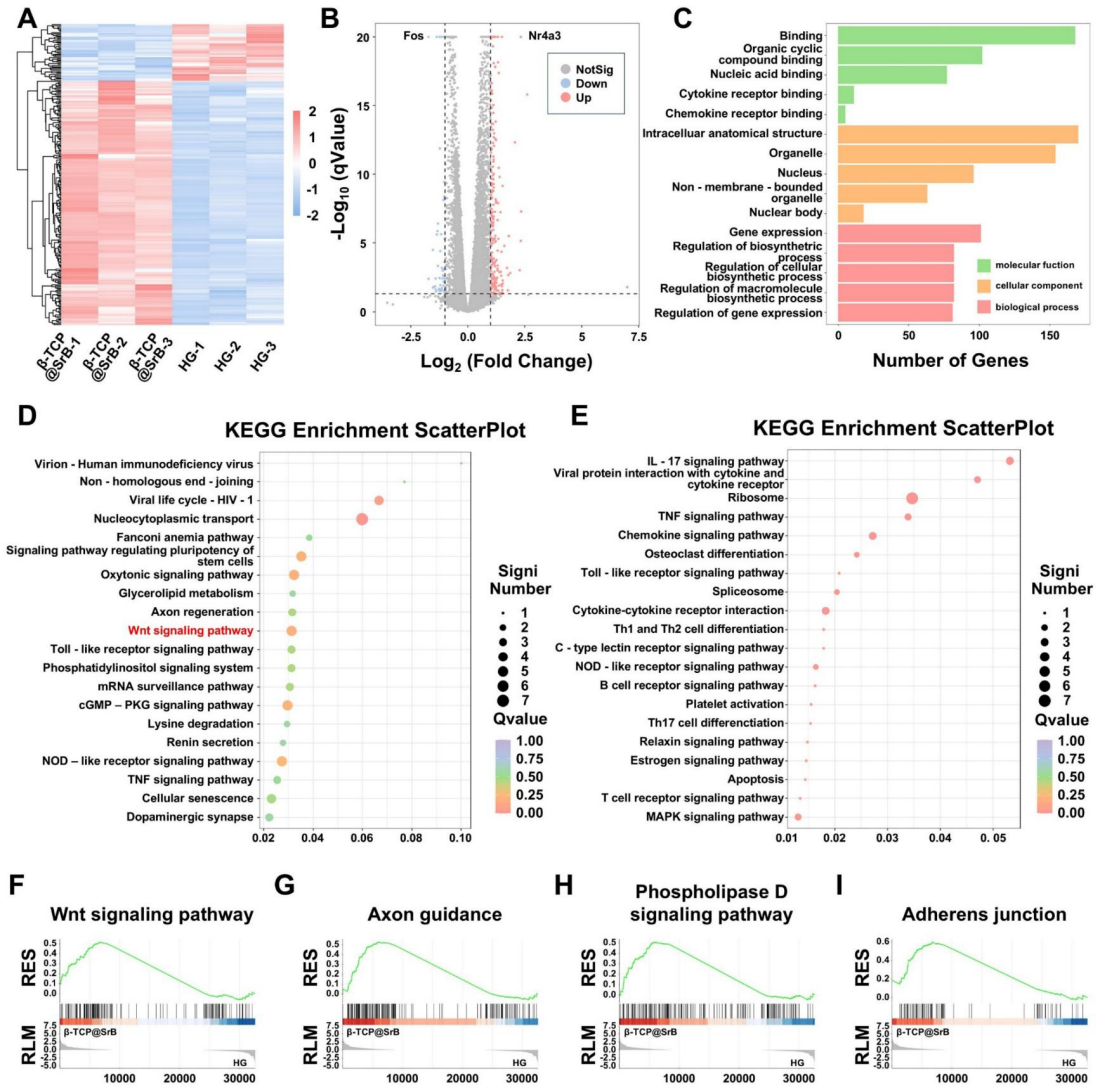
RNA-seq analysis of HG-impaired BMSCs in response to β-TCP@SrB treatment. (**A**) Hierarchical clustering heatmap of differentially expressed genes in HG-treated BMSCs with or without exposure to β-TCP@SrB. (**B**) Volcano plot of significantly altered genes between the two experimental groups. (**C**) Functional enrichment of DEGs based on Gene Ontology analysis. (**D**) KEGG analysis showing the 20 most enriched upregulated pathways, with selected key pathways marked in red. (**E**) KEGG analysis showing the 20 most enriched downregulated pathways. (**F–I**) Gene set enrichment plots for Wnt signaling, axon guidance, phospholipase D signaling, and adherens junction, respectively.

To further elucidate the molecular mechanisms underlying the pro-osteogenic effects of the SrB coating under hyperglycemic conditions, transcriptomic profiling was performed on BMSCs treated with or without β-TCP@SrB under HG conditions. Principal component analysis (PCA) demonstrated obvious divergence between the two experimental groups on the first principal component, indicating extensive gene-expression remodeling after β-TCP@SrB treatment in HG-challenged BMSCs (Supplementary Figure S10). Consistently, hierarchical clustering and heatmap analysis demonstrated high intra-group consistency and pronounced inter-group differences in gene expression patterns, with multiple gene clusters exhibiting coordinated regulation in response to β-TCP@SrB treatment (Figure 5A). Volcano plot analysis identified a set of significantly differentially expressed genes (DEGs) using a stringent threshold of |log_2_(fold change)| > 1 and *q* < 0.05 (Figure 5B). Compared with HG-treated cells, β-TCP@SrB treatment resulted in 216 genes with increased expression and 50 genes with reduced expression (Supplementary Figure S11). Notably, the stress-responsive gene Fos was strongly suppressed (log_2_FC = −1.72, *q* = 3.45 × 10^−75^). Fos encodes a core component of the AP-1 transcriptional complex and is known to promote apoptosis and inhibit Runx2 activity under oxidative stress conditions. In contrast, the nuclear receptor gene Nr4a3 was significantly upregulated (log_2_FC = 2.32, *q* = 3.11 × 10^−25^), which is reported to enhance endogenous antioxidant defenses through activation of the Nrf2 pathway.

GO analysis was used to characterize the functional relevance of the identified DEGs (Figure 5C). In the biological process domain, the most prominently represented terms were ‘gene expression’, ‘regulation of biosynthetic process’, ‘regulation of cellular biosynthetic process’, ‘regulation of macromolecule biosynthetic process’ and ‘regulation of gene expression’. These enrichments indicate that β-TCP@SrB treatment is closely associated with modulation of fundamental cellular processes related to transcriptional regulation, protein biosynthesis and metabolic activity. Kyoto Encyclopedia of Genes and Genomes (KEGG) pathway analysis further identified the key signaling networks regulated by β-TCP@SrB treatment. Among the upregulated pathways, the ‘Wnt signaling pathway’ was prominently enriched and is widely recognized as a canonical regulator of osteogenic differentiation through stabilization of β-catenin and activation of RUNX2-dependent transcription (Figure 5D). In contrast, several pathways were significantly downregulated, including the ‘IL-17 signaling pathway’ and ‘osteoclast differentiation’. Suppression of the ‘IL-17 signaling pathway’ is consistent with attenuation of chronic inflammation under sustained oxidative stress, whereas downregulation of ‘osteoclast differentiation’ suggests reduced osteoclastogenic signaling and maintenance of bone homeostasis (Figure 5E). Gene set enrichment analysis (GSEA) further confirmed significant positive enrichment of the ‘Wnt signaling pathway’, as well as additional pathways including ‘axon guidance’, ‘phospholipase D signaling pathway’, and ‘adherens junction’ (Figure 5F–I).

Overall, these transcriptomic analyses demonstrate the osteoinductive potential of the β-TCP@SrB scaffold and suggest that coordinated regulation of osteogenesis-related signaling pathways, particularly the ‘Wnt signaling pathway’, may underlie its biological effects.

### SrB modified β-TCP promoted bone repair in a T2DM defect model

**Figure 6:**
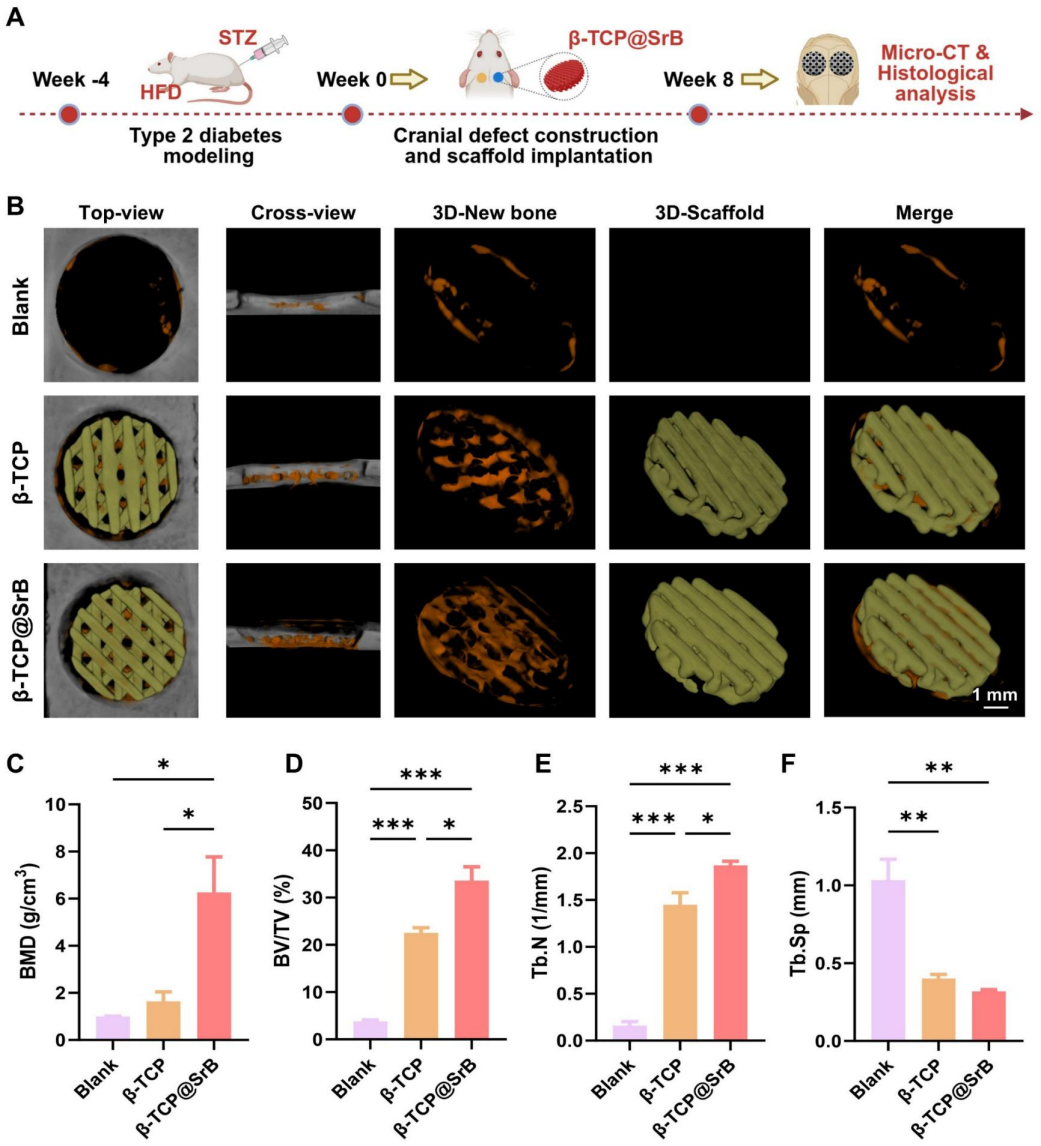
Evaluation of bone repair by β-TCP@SrB scaffolds in T2DM rats. (**A**) Overview of the experimental schedule. (**B**) Representative 3D micro-CT reconstructions of calvarial defects implanted with β-TCP or β-TCP@SrB. Orange indicates newly regenerated bone, and yellow represents the residual scaffold. (**C–F**) Quantitative micro-CT parameters used to assess bone regeneration in the defect region, including BMD, BV/TV, Tb.N and Tb.Sp. *n* = 3. Panel A was prepared using BioRender.com.

The *in vivo* bone-repair capacity of β-TCP@SrB was subsequently investigated in a T2DM rat calvarial defect model after its pro-osteogenic effect had been verified under high-glucose conditions *in vitro*. Critical-sized calvarial defects were filled with either β-TCP or β-TCP@SrB. The bone regeneration performance was evaluated at 8 weeks after implantation (Figure 6A). Micro-CT analysis showed only limited bone regeneration along the edges of the defect in the Blank group, reflecting poor healing in the diabetic setting (Figure 6B). Relative to empty defects, both implanted materials supported bone ingrowth within the defect area. However, the response obtained with β-TCP@SrB was more pronounced than that achieved with β-TCP alone. This difference was also reflected in the quantitative micro-CT data, which indicated that β-TCP@SrB produced the most effective bone repair among the tested groups. More detailed analysis showed that the β-TCP@SrB group achieved the most favorable micro-CT outcomes, with increased BMD, BV/TV and Tb.N together with decreased Tb.Sp relative to both the Blank and β-TCP groups (Figure 6C–F). In comparison with the Blank group, BMD, BV/TV and Tb.N obtained from β-TCP@SrB group increased by 6.27-, 33.62- and 1.87-fold, respectively, whereas Tb.Sp decreased to 0.32-fold. Compared with β-TCP alone, the β-TCP@SrB group reached 3.82-, 1.49- and 1.29-fold increases in BMD, BV/TV and Tb.N, respectively, and a 0.79-fold decrease in Tb.Sp.

**Figure 7:**
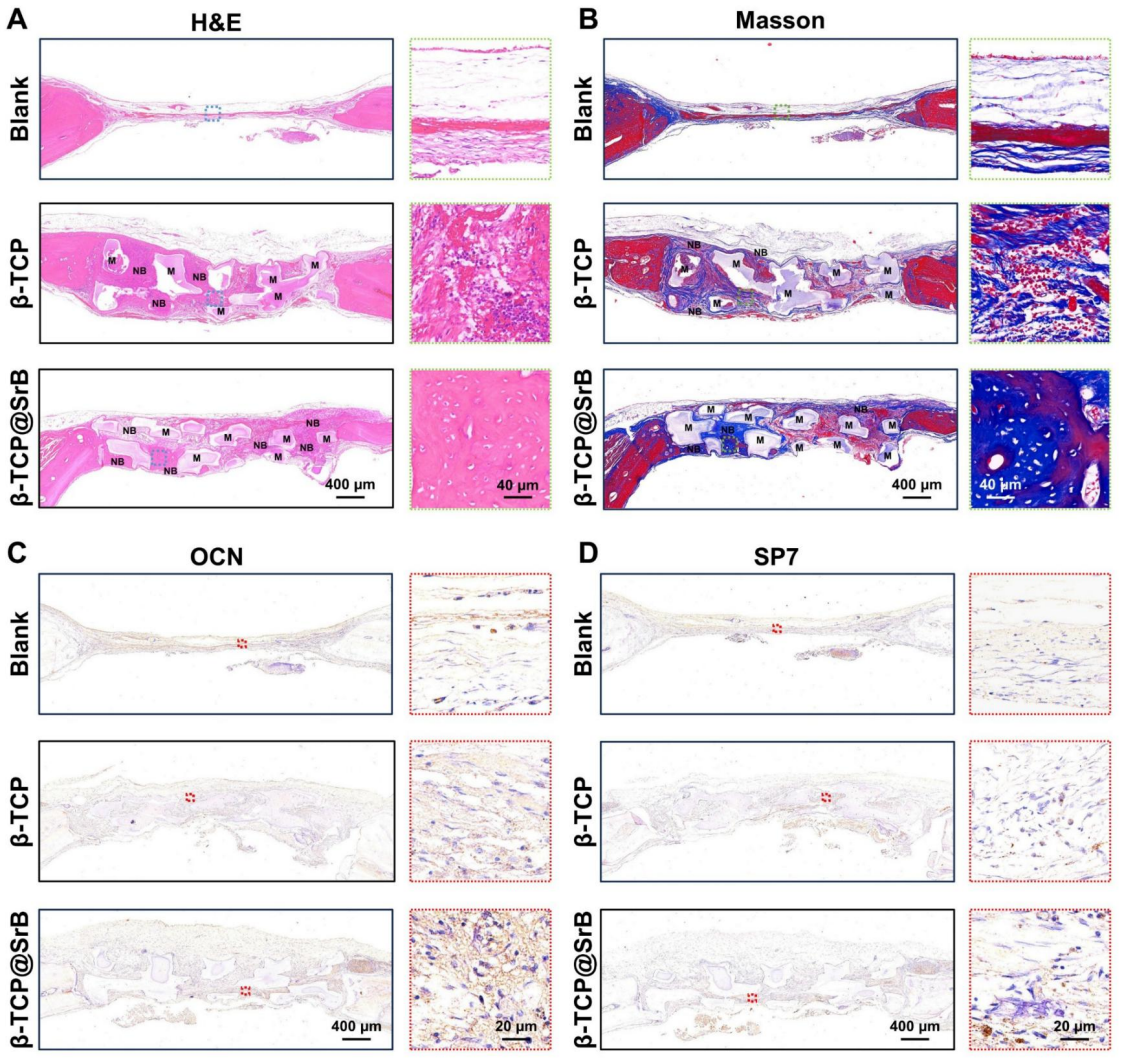
Histological and immunohistochemical evaluation of bone regeneration in diabetic calvarial defects. (**A, B**) Representative H&E staining images (A) and Masson’s trichrome staining images (B) showing newly formed tissue in each treatment group. The implanted scaffold material is denoted as M, and newly formed bone is denoted as NB. Images on the right show higher-magnification views of the corresponding boxed regions. (**C, D**) Immunohistochemical staining images of osteogenic markers of OCN (C) and SP7 (D) in defect regions. Images on the right show higher-magnification views of the corresponding boxed regions.

Further insight into the quality of bone repair was obtained through histological analysis. In the Blank group, diabetic conditions were associated with deficient defect healing on H&E sections, which was manifested by scant newly formed bone and extensive fibrous tissue occupying the defect region (Figure 7A). Both implanted scaffold groups were associated with lower inflammatory cell infiltration and more extensive new bone formation, and this effect was most marked in the β-TCP@SrB group. Masson’s trichrome staining further revealed that β-TCP@SrB scaffolds induced the highest level of collagen deposition and the most mature mineralized bone matrix, indicating superior bone-healing quality and matrix remodeling (Figure 7B).

IHC staining was then used to assess the localization and expression patterns of representative osteogenesis-related markers (Figure 7C and D), including OCN and the transcription factor SP7. In the Blank group, OCN and SP7 signals were weak and sparsely distributed, reflecting suppressed osteoblast activity and limited matrix mineralization under diabetic conditions. In the β-TCP group, OCN and SP7 expression was markedly enhanced, with positive staining broadly localized around newly formed bone tissue and within osteoblast cytoplasm and nuclei, indicating activation of osteogenic differentiation. Notably, the β-TCP@SrB group exhibited the strongest OCN and SP7 staining intensity and the highest proportion of positive cells among all groups, with signals localized to regions of new bone formation, reflecting superior osteogenic induction and regenerative potential.

Taken together, the animal data suggest that SrB surface modification strengthened the bone-repair capacity of β-TCP under diabetic conditions and was associated with accelerated healing and superior regeneration quality.

## Discussion

Diabetic bone defects present a fundamentally different regenerative challenge compared with non-diabetic conditions, as hyperglycemia induces a persistent pathological microenvironment characterized by excessive oxidative stress, chronic inflammation and metabolic dysregulation [14, 29]. Taken together, these disturbances impair osteogenic differentiation, favor osteoclast-mediated bone resorption, and disturb extracellular matrix remodeling, making conventional osteoconductive scaffolds inadequate for effective repair [30, 31]. A growing body of work has explored strategies to address diabetic bone regeneration by either enhancing osteogenic signaling, such as through Sr-doped calcium phosphate ceramics, or mitigating oxidative stress using antioxidant materials or ROS-scavenging nanostructures [32, 33]. However, these strategies often yield limited therapeutic efficacy when applied in isolation, as they target downstream outcomes rather than the upstream pathological constraints imposed by the diabetic microenvironment [34]. In this study, the SrB coated β-TCP scaffold demonstrates that effective bone regeneration in diabetes requires an integrated approach that simultaneously alleviates microenvironmental stress and reactivates osteogenic programs. Rather than functioning as a passive bone substitute, the SrB coating transforms the scaffold into a pathology-adaptive biointerface capable of actively remodeling the diabetic niche to restore regenerative competence.

An important design feature of this work is the interface-confined coupling of antioxidative and osteogenic activities by means of a coordination-driven metal–polyphenol network [35]. Existing Sr-modified biomaterials primarily rely on bulk doping or ionic substitution to deliver osteogenic cues, which may be insufficient under conditions where oxidative stress actively suppresses RUNX2 activity and mitochondrial function [36]. Conversely, antioxidant-based approaches, such as polyphenol-loaded scaffolds or ROS-scavenging nanoparticles, can effectively reduce oxidative damage but often lack sustained osteoinductive capacity, resulting in incomplete or transient regeneration [37]. The SrB coating bridges this gap by confining both functionalities to the material-tissue interface, where pathological signals are most intense. Coordination between Sr^2+^ ions and baicalein provides stable surface anchoring, strong interfacial attachment, and adjustable coating thickness without altering the bulk mechanical or structural characteristics of the β-TCP scaffold. From an engineering perspective, this strategy offers distinct advantages over conventional coatings: mild aqueous processing, strong substrate affinity, modular tunability, and compatibility with clinically established calcium phosphate implants. These features collectively position the SrB coating not merely as a material modification, but as a generalizable interface-engineering platform for pathology-driven tissue regeneration.

At the mechanistic level, the osteogenic efficacy of the SrB coating can be attributed to the complementary yet functionally distinct biological roles of baicalein and Sr^2+^ ions, which together address key pathological constraints imposed by the diabetic microenvironment. Hyperglycemia-associated oxidative stress is widely recognized as a dominant upstream inhibitor of osteogenic differentiation, primarily through disruption of mitochondrial homeostasis, suppression of RUNX2 activity, and induction of stress-responsive signaling that favors apoptosis over differentiation [5, 6]. As a flavonoid polyphenol with established antioxidant and cell-protective effects, baicalein has been reported to attenuate ROS accumulation and strengthen endogenous redox homeostasis, thereby protecting osteogenic cells against oxidative stress-induced damage [38, 39]. Under diabetic bone-healing conditions, such redox regulation is particularly critical, as excessive ROS not only damages cellular components but also actively blocks osteogenic signaling cascades [10]. By attenuating oxidative stress at the material-cell interface, baicalein relieves this inhibitory barrier, thereby creating a permissive intracellular environment in which osteogenic programs can be reactivated.

In parallel, Sr^2+^ ions provide a distinct osteoinductive stimulus that operates through mechanisms largely independent of direct antioxidant activity [40]. Extensive prior studies have demonstrated that Sr^2+^ can activate calcium-sensing receptors on osteoblast-lineage cells, leading to downstream signaling that promotes osteogenic differentiation, matrix synthesis, and mineralization, while simultaneously suppressing osteoclast formation and activity [28, 41, 42]. However, under diabetic conditions, the efficacy of Sr^2+^ alone may be compromised, as oxidative stress and inflammatory signaling interfere with osteogenic transcriptional machinery and energy metabolism [5]. The present results suggest that baicalein-mediated redox regulation enables Sr-driven osteogenic signaling to function more effectively under hyperglycemic stress. In this coordinated system, baicalein primarily acts to restore cellular resilience and metabolic competence, whereas Sr^2+^ serves as an instructive cue directing lineage commitment and matrix formation. This functional division provides a mechanistic basis for the observed synergy between baicalein and Sr, whereby antioxidant protection and osteogenic induction are spatially and temporally integrated at the scaffold interface to overcome diabetes-associated suppression of bone formation.

Importantly, the coordination assembly of Sr^2+^ and baicalein into a metal-polyphenol network is not merely a delivery vehicle but plays an active role in sustaining their cooperative biological effects. Surface-confined coordination stabilizes baicalein against rapid diffusion or degradation while enabling localized Sr^2+^ presentation, ensuring that both components act predominantly at the bone-material interface where pathological signals are most pronounced. Such interface-localized coordination may be particularly advantageous in diabetic environments, where systemic delivery of antioxidants or osteogenic agents often suffers from limited targeting efficiency and off-target effects. By integrating antioxidative protection with multiple modes of osteogenic regulation, namely redox-dependent rescue of osteoblast function and Sr^2+^-mediated activation of osteogenic signaling, the SrB coating establishes a multifunctional biointerface capable of addressing the complex, multifactorial nature of diabetic bone repair failure.

Notably, oxidative stress, chronic inflammation and bone metabolic imbalance represent common pathological features across multiple orthopedic disorders, suggesting that the dual antioxidative and osteogenic functions of SrB may have broader therapeutic potential. In osteoporotic bone defects, where excessive osteoclast activity and impaired osteogenesis are accompanied by systemic oxidative stress, Sr^2+^-mediated bone regulation combined with baicalein-driven ROS scavenging may synergistically restore bone homeostasis [43]. In inflammatory conditions such as chronic osteomyelitis and peri-implantitis, β-TCP@SrB may act as a local anti-inflammatory and pro-regenerative material by suppressing oxidative damage while promoting new bone formation [44, 45]. Similarly, in radiation-induced bone injury and post-extraction alveolar bone loss, where persistent ROS and vascular impairment hinder regeneration, the sustained release of Sr^2+^ and baicalein may provide continuous microenvironmental modulation to support tissue repair. Collectively, these findings indicate that β-TCP@SrB may serve as a versatile, locally delivered therapeutic platform for a range of bone-related diseases [46, 47].

Although the current findings are encouraging, the study still has several limitations that should be recognized. First, although the combined antioxidative and osteogenic effects of the SrB coating are supported by both functional and transcriptional evidence, the precise molecular interactions linking redox regulation to specific osteogenic signaling nodes remain incompletely defined and warrant further mechanistic investigation. Second, even though short-term *in vivo* data indicate better bone healing in diabetic animals, the long-term fate, stability, and potential systemic effects of SrB coatings, particularly with respect to prolonged Sr^2+^ release, require extended evaluation in chronic models. Third, the current study focuses on a single diabetic bone defect model, and whether the observed benefits can be generalized to other diabetes-associated skeletal complications or to large-animal systems remains to be determined. Addressing these limitations will be essential for translating this interface-engineering strategy into clinically viable therapies.

## Conclusion

In summary, surface modification of β-TCP with a strontium-baicalein coating markedly improved its reparative behavior in diabetic bone defects. By integrating antioxidative protection with osteogenic stimulation at the material-tissue interface, the SrB coating mitigates hyperglycemia induced oxidative stress and restores osteogenic differentiation. In a diabetic rat model, SrB modified scaffolds promoted both bone formation and bone quality. These results support surface functionalization as an effective means to improve bone regeneration in metabolically impaired settings.

## Supplementary Material

rbag083_Supplementary_Data
