# Supplementary material for: Strontium-baicalein coated β-tricalcium phosphate scaffold enhances diabetic bone regeneration via synergistic ROS scavenging and osteogenic activation
Source: Regen Biomater. 2026 Apr 26;13:rbag083. doi: 10.1093/rb/rbag083 (PMC13198881; doi:10.1093/rb/rbag083)
Supplement: rbag083_Supplementary_Data [file rbag083_supplementary_data.docx]

**Strontium-baicalein coated β-tricalcium phosphate scaffold enhances diabetic bone regeneration via synergistic ROS scavenging and osteogenic activation**

Zhiqiang Liu ^a^ †, Yihao Wu ^b, c^ †, Chen Yang ^c, d *^, Ruqi Wang ^c^, Linyi Hu ^a^, Sitong Hu ^c^, Xitao Wu ^a^, Jiang Chang ^c^, Jianfeng Ma ^b *^, Jiandong Yuan ^a *^

**Supplementary Information**

**Contents of Supplementary Information**

**Figure S1** Solubility of baicalein in aqueous solution at different pH values.

**Figure S2** Formation of SrB coatings on the surface of β-TCP scaffolds at different Sr-baicalein molar ratios.

**Figure S3** Formation of SrB coatings on the surface of β-TCP scaffolds at different coating cycles.

**Figure S4** Viability of BMSCs cultured with β-TCP@SrB scaffolds prepared using different numbers of coating cycles for 24 h. *n = 5.*

**Figure S5** Schematic diagram of the coordination structure between Sr²⁺ and baicalein

**Figure S6**: UV-Vis absorption spectra of baicalein and SrB complexes.

**Figure S7**: In vitro degradation profiles of β-TCP and β-TCP@SrB scaffolds over 14 days. *n = 5*.

**Figure S8:** Representative images of ABTs solutions after reaction with β-TCP and β-TCP@SrB scaffolds..

**Figure S9:** Representative images of DPPH solutions after reaction with β-TCP and β-TCP@SrB scaffolds.

**Figure S10:** PCA plot of differentially expressed genes (DEGs) in the β-TCP@SrB and HG groups.

**Figure S11:** Quantification of differentially expressed genes (DEGs) between the β-TCP@SrB and HG groups.


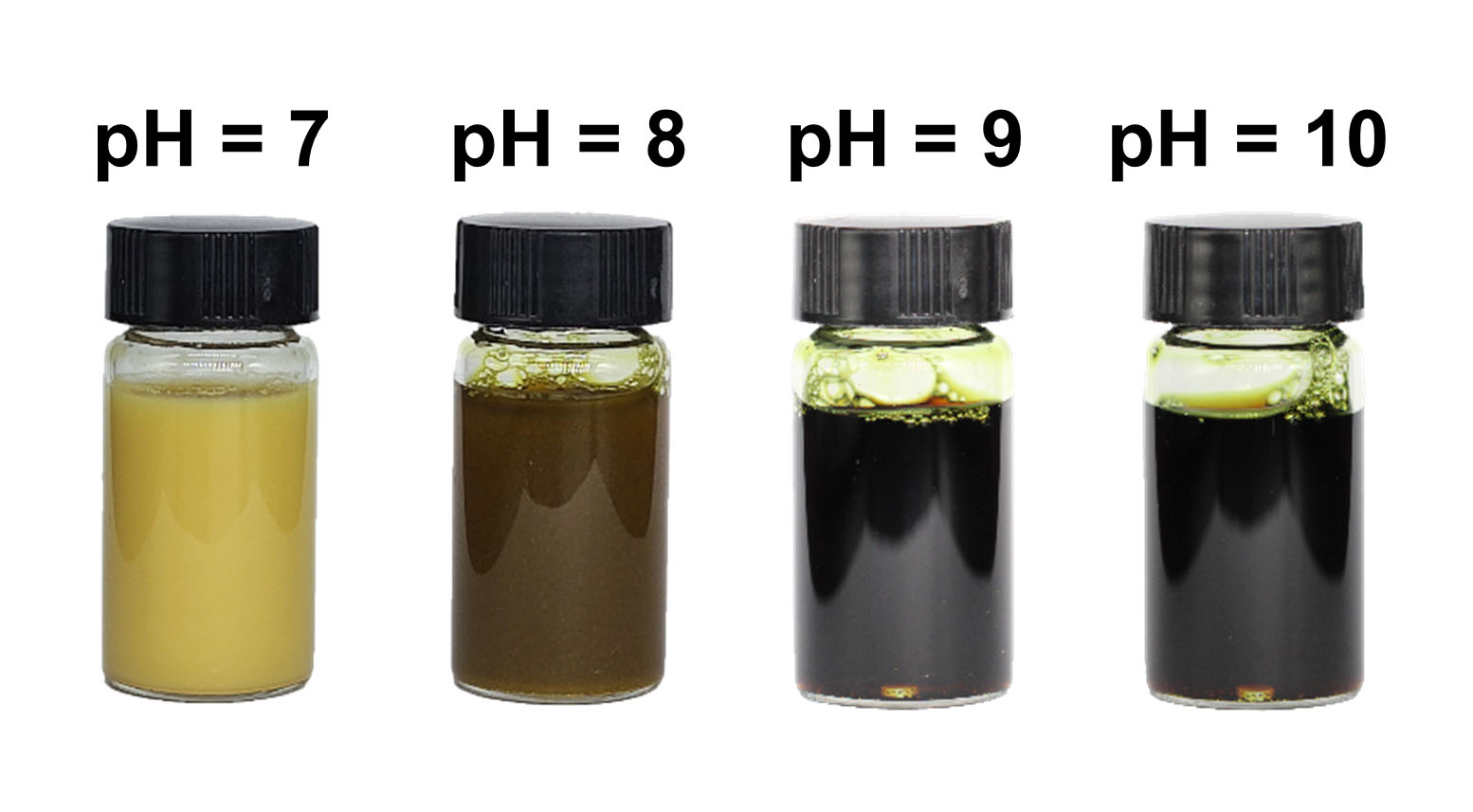


**Figure S1** Solubility of baicalein in aqueous solution at different pH values.


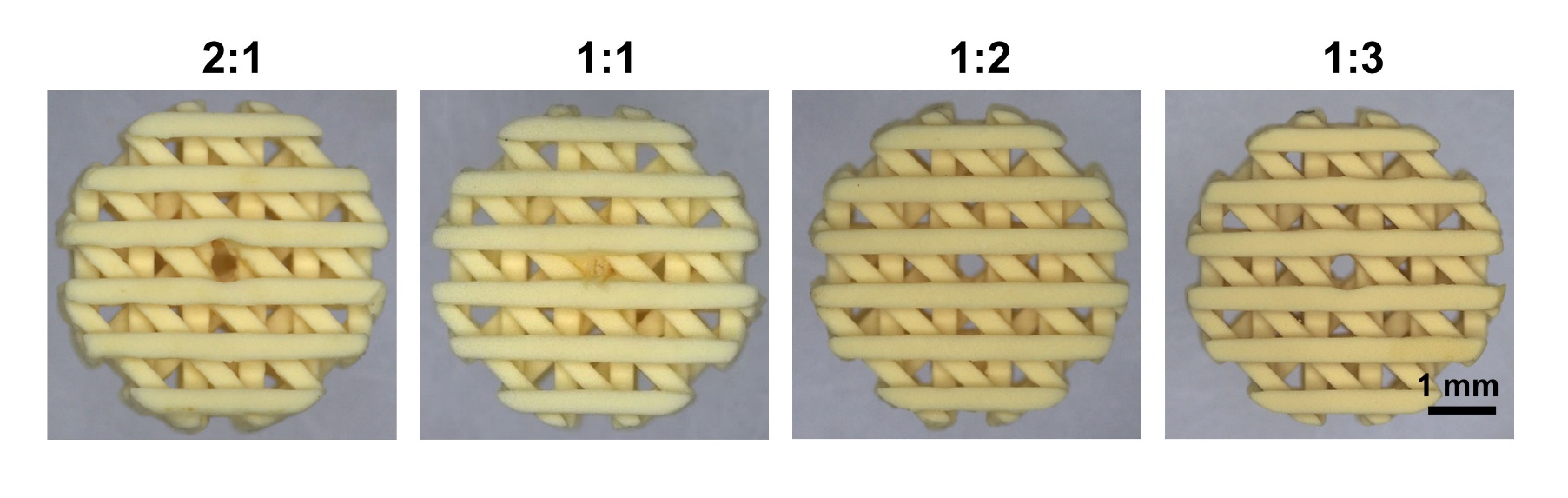


**Figure S2** Formation of SrB coatings on the surface of β-TCP scaffolds at different Sr-baicalein molar ratios.


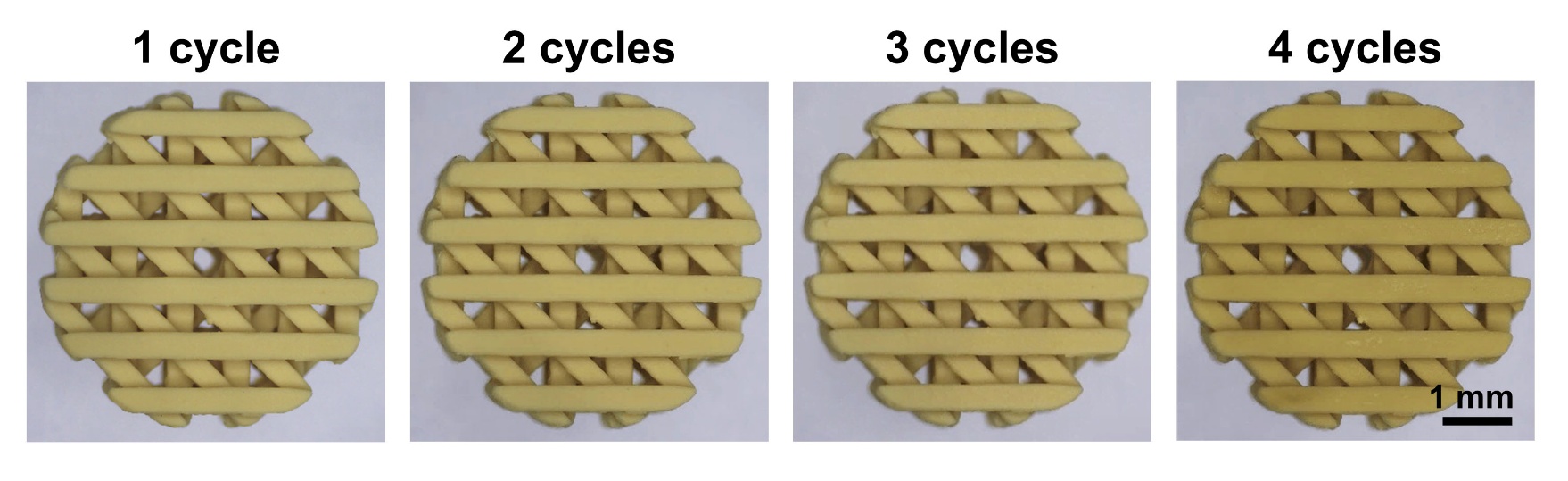


**Figure S3** Formation of SrB coatings on the surface of β-TCP scaffolds at different coating cycles.


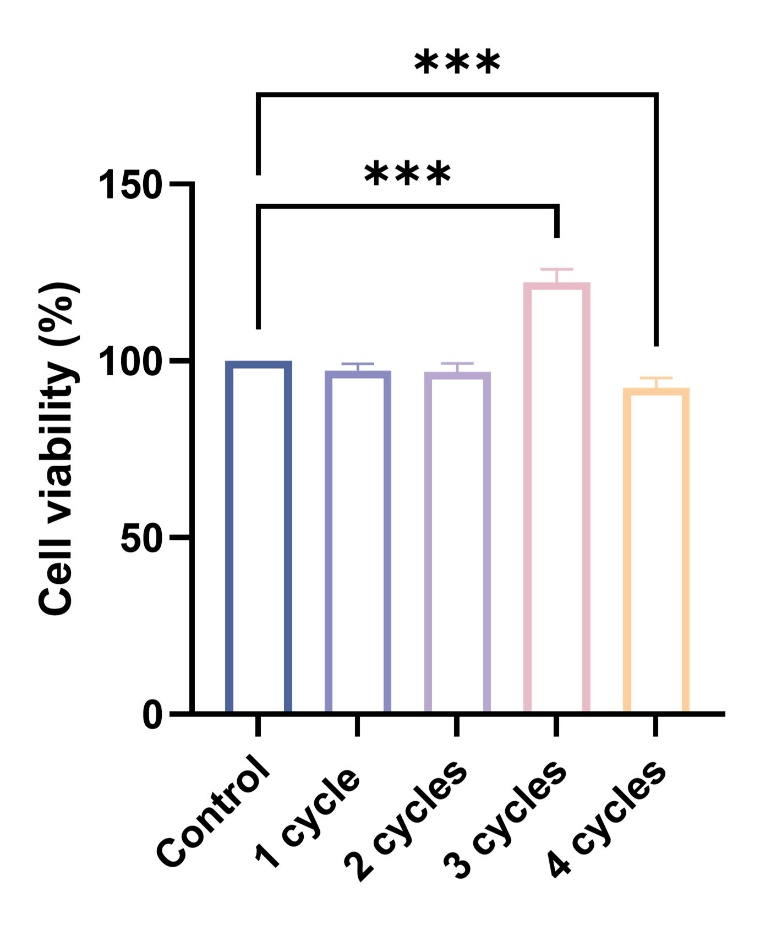


**Figure S4** Viability of BMSCs cultured with β-TCP@SrB scaffolds prepared using different numbers of coating cycles for 24 h. *n = 5.*


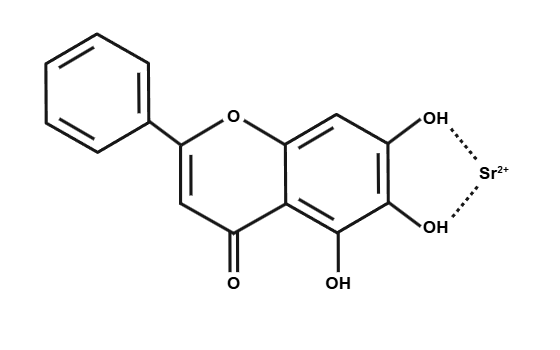


**Figure S5** Schematic diagram of the coordination structure between Sr²⁺ and baicalein


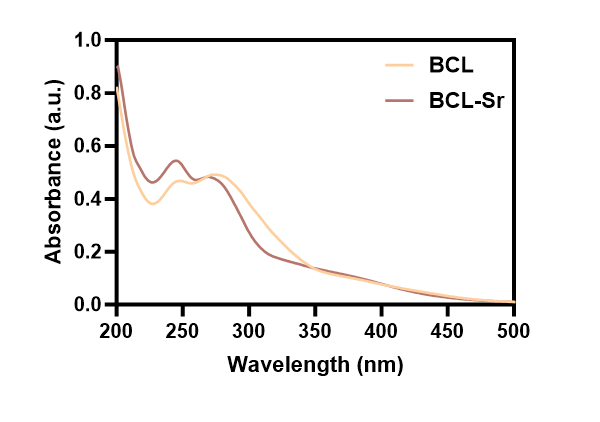


**Figure S6.** UV-Vis absorption spectra of baicalein and SrB complexes.


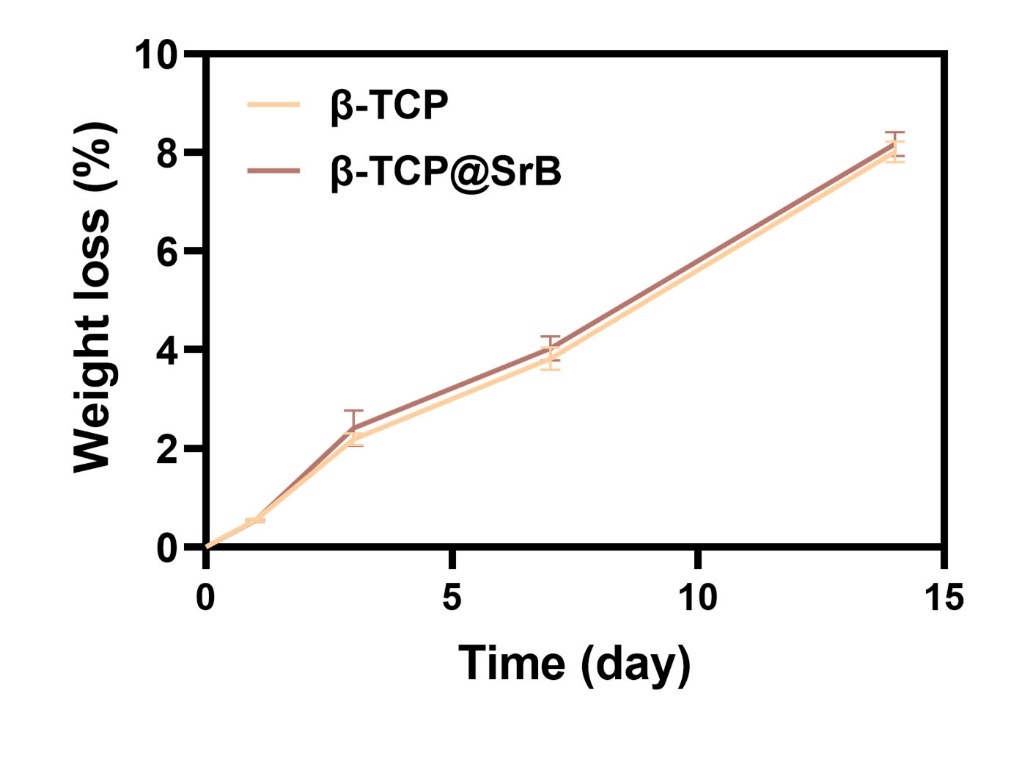


**Figure S7.** In vitro degradation profiles of β-TCP and β-TCP@SrB scaffolds over 14 days. *n = 5*.


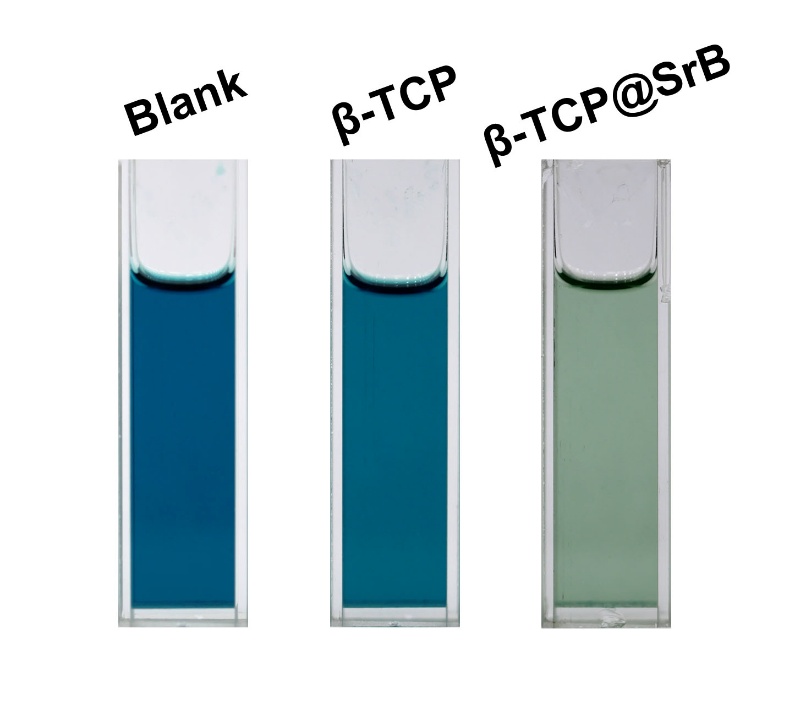


**Figure S8.** Representative images of ABTs solutions after reaction with β-TCP and β-TCP@SrB scaffolds.


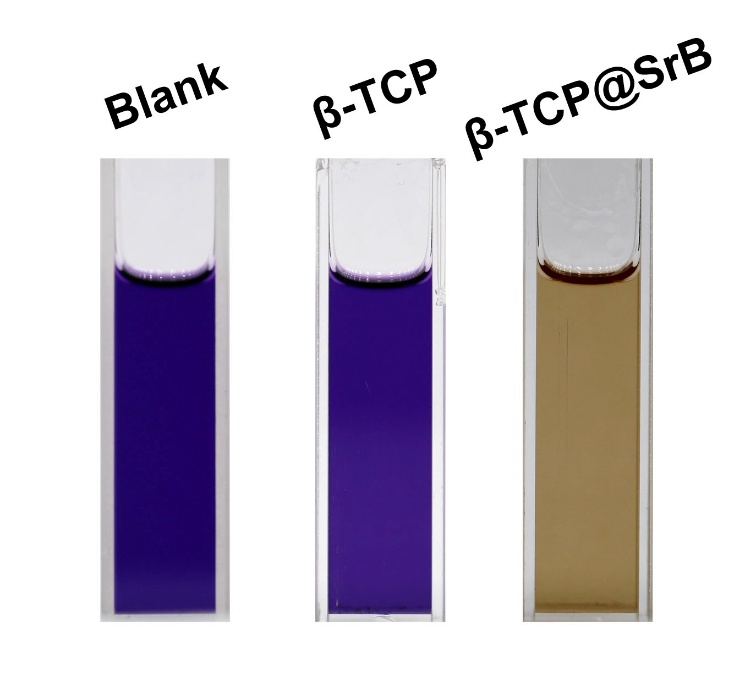


**Figure S9.** Representative images of DPPH solutions after reaction with β-TCP and β-TCP@SrB scaffolds.


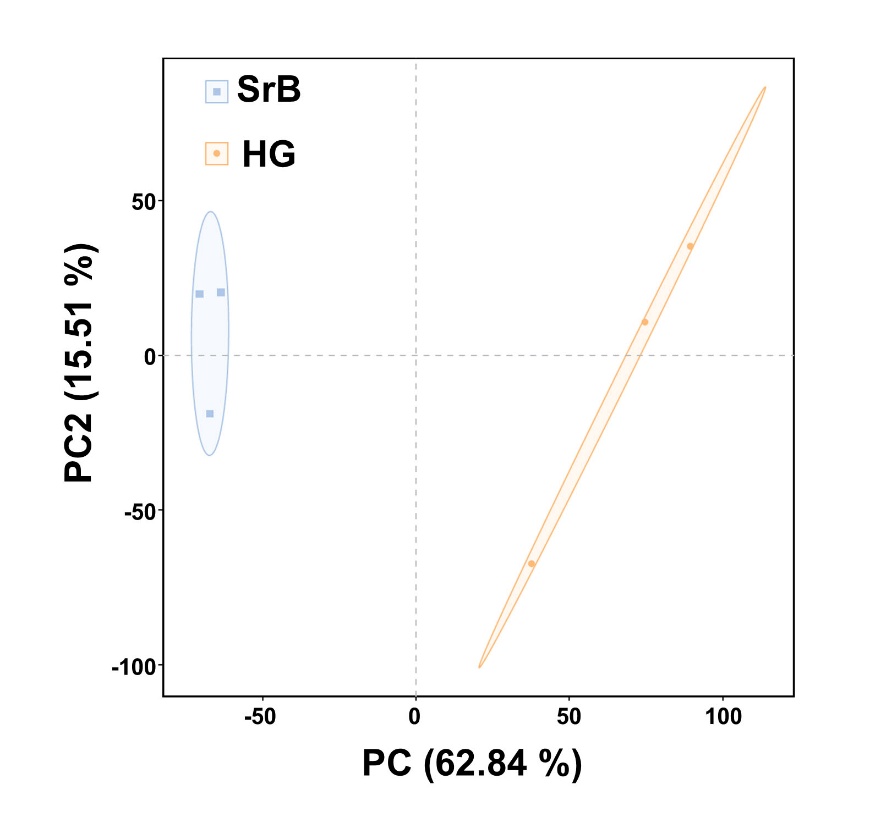


**Figure S10.** PCA plot of differentially expressed genes (DEGs) in the β-TCP@SrB and HG groups.


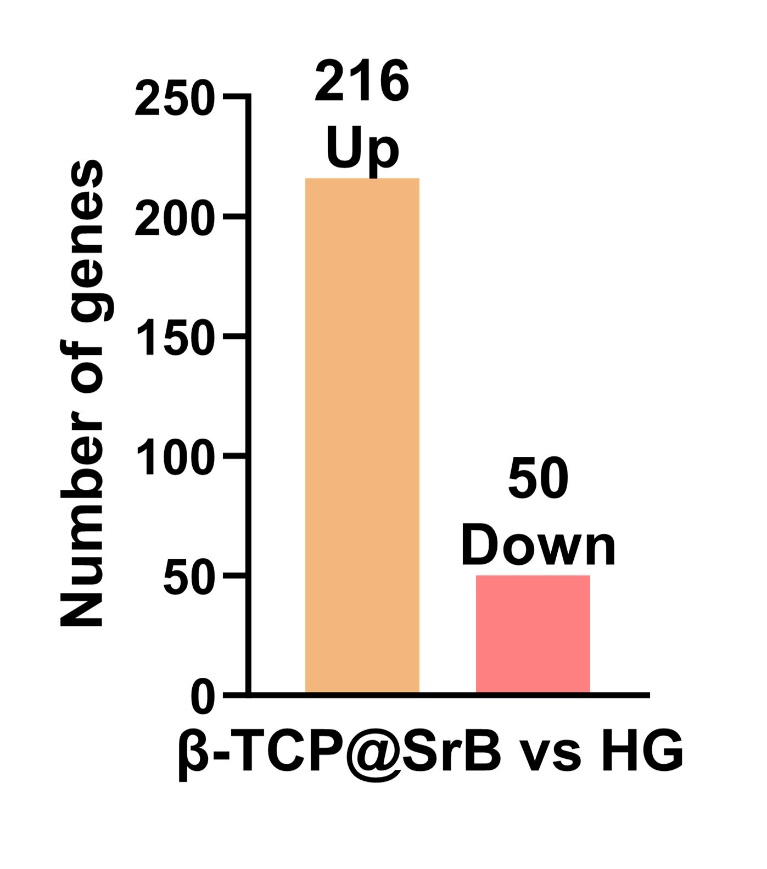


**Figure S11.** Quantification of differentially expressed genes (DEGs) between the β-TCP@SrB and HG groups.
